# Patient education for individuals with Interstitial Lung Disease: A scoping review.

**DOI:** 10.12688/f1000research.147340.1

**Published:** 2024-04-26

**Authors:** Revati Amin, K. Vaishali, G. Arun Maiya, Aswini Kumar Mohapatra, Mukesh Kumar Sinha, Anup Bhat, Tulasiram Bommasamudram, Vishak Acharya, Shweta Gore

**Affiliations:** 1 Physiotherapy, Manipal College of Health Professions, Manipal Academy of Higher Education, Manipal, Karnataka, 576104, India; 2 Respiratory Medicine, Kasturba Medical College, Manipal Academy of Higher Education, Manipal, Karnataka, 576104, India; 3 Exercise and Sports Science, Manipal College of Health Professions, Manipal Academy of Higher Education, Manipal, Karnataka, 576104, India; 4 Pulmonary Medicine, Kasturba Medical College, Manipal Academy of Higher Education, Mangalore, Karnataka, 575001, India; 5 MGH Institute of Health Professions, Boston, Massachusetts, 02129, USA

**Keywords:** educational contents, effectiveness, interstitial lung disease, patient education, process of delivery, pulmonary rehabilitation, scoping review

## Abstract

**Objectives:**

Interstitial Lung Disease (ILD) is a severe and rapidly progressing disease with a high fatality rate. Patient education (PE) has been demonstrated to promote long-term adherence to exercise and lifestyle improvements by assisting patients in developing self-management techniques. Our scoping review's goal was to chart out the prevailing level of research about the content, processes, and effectiveness of PE for patients with ILD.

**Methods:**

The relevant databases were searched using the rules provided by Arksey and O'Malley in 2005 and the Joanna Briggs Institute reviewers' manual 2015: an approach for JBI scoping reviews. Individuals with ILD, published in English between the years of inception and 2020, and describing PE administered by various healthcare practitioners were among the 355 studies found and reviewed. Thirteen studies met these criteria.

**Results:**

PE delivery process, delivery techniques, quality of life assessments, common PE themes, and healthcare professional participation were all recognized and cataloged.

**Conclusion:**

Despite the fact that healthcare professionals (physicians, nurses, and physiotherapists) provide PE to patients with ILD regularly, the PE provided varies greatly (contents of PE, process of delivery and delivery techniques). During the scoping review, a significant variation in the themes was addressed. They could not provide any evidence-based specific recommendations for all healthcare practitioners due to the studies' heterogeneity and lack of effectiveness measures.

**Abbreviations**
ATAQ IPF: A Tool to Assess Quality of Life in Idiopathic Pulmonary Fibrosis
BDI: Beck’s Depression Inventory
chILD QoL: child Interstitial Lung Disease Quality of Life
COPD: Chronic Obstructive Pulmonary Disease
CRQ: Chronic Respiratory Questionnaire
DLCO: Diffusing capacity of lung of carbon monoxide
FEV1: Forced Expiratory Reserve Volume in 1 second
FVC: Forced Vital Capacity
IPF: Idiopathic Pulmonary Fibrosis
ILD: Interstitial Lung Disease
IPAQ: International Physical Activity Questionnaire
KBILD: King’s brief Interstitial Lung Disease
MBS: Modified Borg Scale
PE: Patient Education
PR: Pulmonary Rehabilitation
QOL: Quality of Life
SF-36: Short Form- 36
SGRQ: St. George Respiratory Questionnaire
UCSD SOBQ: University of California, Shortness of Breath Questionnaire
6 MWD: 6 Minute Walk Distance

## 1. Introduction

Interstitial lung disease (ILD) is a condition that leads to an incremental inability to maintain normal levels of blood oxygen due to impaired gas exchange through the alveolar-capillary membrane. ^1^ ILD is frequently associated with poor health outcomes including exercise limitation, exercise-induced breathlessness, decreased physical activity, poor quality of life, increased health service utilization, and death. ^2^^–^^6^ The experience with ILD is that of a long way to diagnosis, intense symptomatic burden, insufficient information of the disease and anxiety. ^5^

Pulmonary rehabilitation (PR) is a fundamental mode of treatment to improve exercise tolerance and minimize symptoms in patients with chronic lung disease. ^7^^,^^8^ Evidence supports the effectiveness of PR in ILD and guidelines for the management of ILD have adopted PR as a priority intervention. PR encompasses a range of interventions focussed on the individual needs of the patient. One important component of PR is Patient Education (PE).

PE helps patients develop self-management strategies and has shown to improve long-term adherence to exercise and lifestyle changes. PE offers insight into the knowledge that patients need for the best possible treatment for chronic diseases. ^9^ The educational programs seek to strengthen disease management ^10^^–^^15^ and enhance self-efficacy. ^16^^,^^17^ Various guidelines generated by national and international advisory committees emphasize the value of PE in chronic lung disease. ^18^^–^^21^ The instructional content should be readily available and strategically focused on providing efficient, cost-effective, and high-quality health education. Quality education in health seeks to enhance the lives of individuals through the encouragement of improvement in attitudes, values, awareness, skills, and behaviour. ^22^ Oxygen therapy, exacerbation control, energy conservation, symptom management, mood disorders, medications, lung transplants, and end-of-life care are all topics covered in educational programs for ILD patients. ^23^

PE can be broken down into five distinct phases. The patient's prior knowledge, misconceptions, learning abilities, cognition, comprehension, attitudes, and motivation are all assessed in the first stage. Following the assessment, the patient's approaches, problems, and learning needs will be diagnosed. The third phase is to work with the patient to organize education, set goals, and choose educational interventions. It is critical to discuss the type of education, the frequency, who will offer the education, and when and how it will be addressed during the planning process.

Scoping reviews analyze the scope and depth of available data on a particular topic, as well as the nature of published studies, in order to provide a comprehensive overview of broad, varied literature. Scoping reviews also allow researchers to go further into specific research topics in order to conduct more thorough systematic reviews. When time is restricted and there is no clear information or knowledge about a topic to start a systematic review, a scoping review is undertaken.

A continuing assessment of patient needs and priorities provides the framework for further education. Since PR was developed and designated for patients with chronic obstructive pulmonary disease (COPD), which has a disease mechanism and symptoms that are fundamentally different from ILD. PE issues defined for patients with COPD, such as the physiological basis for exercise limitation, symptoms, treatment, disease severity, and outcomes, may not be relevant or significant for patients with other pulmonary disorders, such as ILD. Although healthcare practitioners routinely deliver PE verbally to patients with ILD, little is known about the actual content and mechanisms of this provision, as well as its overall efficacy. There is a need to map the available research because there is a lack of evidence about the administration and content of PE delivered in ILD patients. Therefore, the goal of this scoping review was to define and map the current information of the content, processes, and efficiency of PE provided for patients with ILD.

## 2. Materials and methods

The methodology framework used in this study was initially outlined by Arksey and O’Malley ^24^ and revised Joanna Briggs Institute reviewers' manual 2015: methodology for JBI scoping reviews. ^25^ The scoping review is reported in accordance with the Preferred Reporting Items for Systematic Reviews and Meta-Analyses statement for reporting scoping reviews (PRISMA-ScR). ^26^ The framework was divided into five independent phases: formulating the research question; locating relevant studies; identifying studies; organizing and extracting data; and, at last, summarizing and presenting the results.

### Formulating the research questions

The specific research questions were as follows:

1. What are the contents (topics) of the PE provided for patients living with ILD?
2. What processes or methods were used when providing PE to patients living with ILD?

### Locating relevant studies

We undertook a comprehensive PubMed and Scopus literature search to identify studies describing the PE provided for patients with ILD. Inclusion criteria consisted of studies including patients with ILD (aged > 18 years) referred for PR in any health care setting. The PR sessions have to be inclusive of PE administration using either booklets, leaflets, websites, mobile applications or verbal/practical demonstration. Articles published in English from inception to 2020. The content of the PE (topics addressed, different processes of the PE intervention, location of delivery, mode, who delivered PE, and technique of delivery), provided through a pulmonary rehabilitation program. Types of sources: Any type of research design, such as quantitative, qualitative, or mixed methods were included in the study. Articles written in languages other than English, editorials and commentaries were excluded.

**Search strategy:** Initially, a PubMed and Scopus search was undertaken, followed by a screening of the titles and abstracts of retrieved papers, as well as the search terms utilized to describe them. A thorough search was then conducted across all of the involved databases utilizing all identified keywords and index terms. In order to locate more pertinent sources, the reference lists of the included papers and reports were examined as well. Grey literature from databases and conference proceedings was also investigated, and duplicate publications were found and eliminated. The titles and abstracts of articles were examined to determine whether they met the inclusion criteria. If an article satisfied the criteria or needed further review before being excluded, its full text was retrieved. A third reviewer (AM) resolved any differences between the two independent reviewers' (RA and VK) assessments of whether the full-text paper fulfilled the inclusion criteria. Using specific terms for each database, the search on PubMed and Scopus concentrated on the three main ideas of ILD, physiotherapy, and patient education (PE).

### Search terms

“Idiopathic pulmonary fibrosis” OR “IPF” OR “pulmonary fibrosis” OR “fibrosis” OR “ILD” OR “interstitial lung disease” OR “lung disease” OR “pulmonary disease” AND “physiotherapy” OR “physical therapy” OR “physical training” OR “physical rehabilitation” OR “pulmonary rehabilitation” OR “pulmonary rehabilitation training” AND “health education” OR “patient care” OR “self-management” OR “health communication” OR “health behaviour” OR “PE” OR “booklets” OR “pamphlets” OR “leaflets” OR “brochures” OR “handout” OR “flyer” OR “electronic educational materials” OR “web-based education” OR “tele-rehabilitation” OR “telerehabilitation” OR “telehealth” OR “telerehab” OR “mHealth” OR “telecare”.

### Identifying the studies

**Figure 1.**
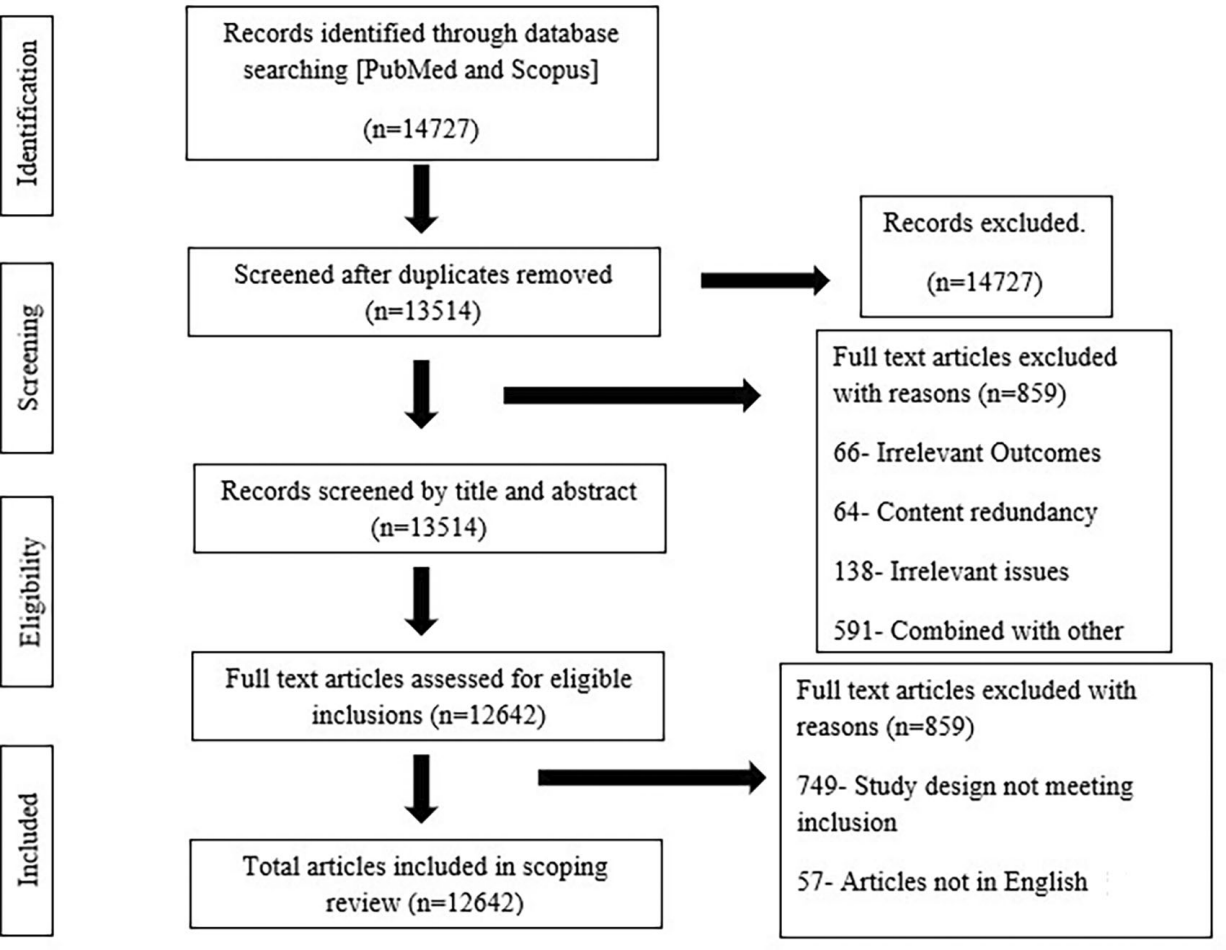
PRISMA Flowchart.

Following the initial screening, 14,727 articles were evaluated through assessing their titles and abstracts against established inclusion and exclusion criteria. When one reviewer (RA) was unsure whether to include or exclude an article, it was independently reviewed by another reviewer (VK). 355 articles were subsequently found to be eligible for full-article review. The 355 papers were then evenly allocated alphabetically between the two reviewers (RA and VK). Each full text article was thoroughly reviewed against the inclusion and exclusion criteria implementing the PRISMA extension for Scoping Reviews (PRISMA ScR) flow approach. ^27^ ( Figure 1)

**Organising and extracting data:** To organize the data collected systematically, a data extraction form was developed. This form consisted of a table with predefined categories that corresponded to the scoping review's objectives and research questions. These categories included information about the author, year of publication, country, study design, sample size, the content of the PE, processes of delivery, effectiveness (outcomes). The data from each article was documented in these specific categories for a thorough analysis. ( Table 1– 3)

**Table 1.** Overview of quantitative study designs.

Sr. No	Author, Year, country	Study design and number	PE tools (method of delivery)	PE Contents described	Effectiveness (outcomes)
1	Niemitz et. al. 2019, Germany	Single group intervention- pre post pilot study, n=107	Patient information brochure available as a web-based resource: http://www.childeu.net	basic education material about the structure and function of airways, chILD -diagnosis, diagnostic procedures, therapy, nutrition, complications, school, leisure time, holidays and traveling, social and financial support, research, self-help groups and a short medical dictionary.	Self-efficacy, satisfaction with PE, chILD-QOL (self-report and caregiver report)
2	Garvey 2010, United states	Systematic Review (2 RCTs, 4 retrospective studies, 1 prospective study)	Recommendation provided: individualized curriculum based on knowledge deficits identified during the initial evaluation.	Mode and associated measures; prevention, recognition, management of exacerbations and pulmonary infections; energy conservation, pacing, management of ADL, symptom anxiety, panic, and mood disorders, medication actions, schedule, side effects, and adherence; lung transplantation and posttransplant rehabilitation; end-of-life care, nutrition.	SGRQ, SF-36
3	Yu et. al. 2019	Systematic Review and Meta-analysis (7 RCTs)	PE included as a part of PR in the studies	No details	SGRQ, SF-36, IPAQ, ATAQ IPF scores, IPF specific SGRQ, BDI, 6MWD, DLCO, PFT
4	Ozalevli et. al. 2010, Turkey	Non-RCT (Prospective study) 17 IPF patients	Booklet	No details	SF-36, MBS- dyspnoea

**Table 2.** Overview of Qualitative study designs.

Sr. No	Author, Year, country	Study design and type	Main contents
1	Holland et. al. 2015, Australia	Qualitative study Individual semi-structured interviews 18 ILD participants	People with ILD (PE components): Telling future-prognosis, honesty from clinicians, end-of-life planning; acceptability of different health professionals, using the internet; ILD specific topics. Clinicians perspective (PE components): Content of PR: specificity to underlying diagnosis, information on current pharmaceutical treatments, Discussing the future in PR: addressing prognosis, end-of-life planning.
2	Holland et. al. 2019, Australia	Qualitative study 2 round Delphi process- focus group discussion	Core education topics: 1. Staying well with 2. Pulmonary rehabilitation 3. Oxygen therapy 4.Managing symptoms (breathlessness and cough fatigue) 5. Managing anxiety, depression and panic Optional education topics: 1. Accessing home care and support for both patients and carers 2. End of life care and advance directives 3. Managing co-existing medical conditions 4. Managing medications and side effects Recommendation: Need for ILD specific PE package
3	Morisset et. al. 2016, United States	Qualitative study Semi-structured interviews for Health care professionals	Multidisciplinary team: Nurse, Pulmonologist, Kinesiologist Patient-Physician-Support groups-Internet PR-group discussion-Trained educator-Psychological support-Content tailored for ILD patients Key educational topics: Pathophysiology of ILD, management of symptoms, clinical tests, autonomy, oxygen use, medications, end-of-life counselling
4	McLean et. al. 2020, Australia	Qualitative study Questionnaire- letters	Requirement of a multidisciplinary team: Respiratory physician, ILD specialist nurse, trials co-ordinator, Rheumatologist, Physiotherapist. Requirement: written educational material. Quality of Life assessment: K-BILD, UCSD SOBQ
5	Ramadurai et. al. 2019, United States	Qualitative study Questionnaire- electronic web-based survey	Pressing needs for requirement of educational materials (printed or internet-based) and more support staff. Needing assistance from family members during interventions. Requirement of more information on lung transplantation, pulmonary rehabilitation programs.

**Table 3.** Overview of Review articles.

Sr. No	Author, Year	Study design	Main contents
1	Quinn et. al. 2019	Review	Web-based layout of PE in ILD: Pulmonary Fibrosis Foundation ( www.pulmonaryfibrosis.org), ( https://www.ofev.com/support/open-doors; https://www.esbriethcp.com/ipf-patient-assistance/patient-education-inspiration.html)
2	Wijsenbeek et. al. 2019	Review	Supportive measures to be provided in ILD: Delivering disease education and psychological support, PR, supplemental O2, ambulatory O2, nocturnal O2 symptom relief: dyspnoea, fatigue, anxiety and depression, cough End-of-life care
3	Wuyts et. al. 2014	Review	Common themes identified: patient-centred care model, the three pillars of care model and the Brompton model of care which include strong patient education, encouraging patient participation and an accessible healthcare team, strong provider–patient relationship. Brompton model of care provides initiatives for simultaneous patient participation and PE.
4	Raghu et. al. 2017	Review	Feelings and perceptions of patients with IPF identifying three emotional phases: coming to terms (diagnosis), reactive coping (acceptance), and proactive coping (ownership of condition) Identified areas of patient concern: physical problem, family support, interactions with the medical world, and hope for research. Delivery of educational and psychological support: including in pulmonary rehabilitation in groups, community-based conferences, patient support groups, and individual counselling. Involvement of Healthcare professionals.

## 3. Results

### Summarizing and presenting the results

The information was collected and summarised for extraction and the results were recorded in the same framework we developed for analysis.

**Figure 2.**
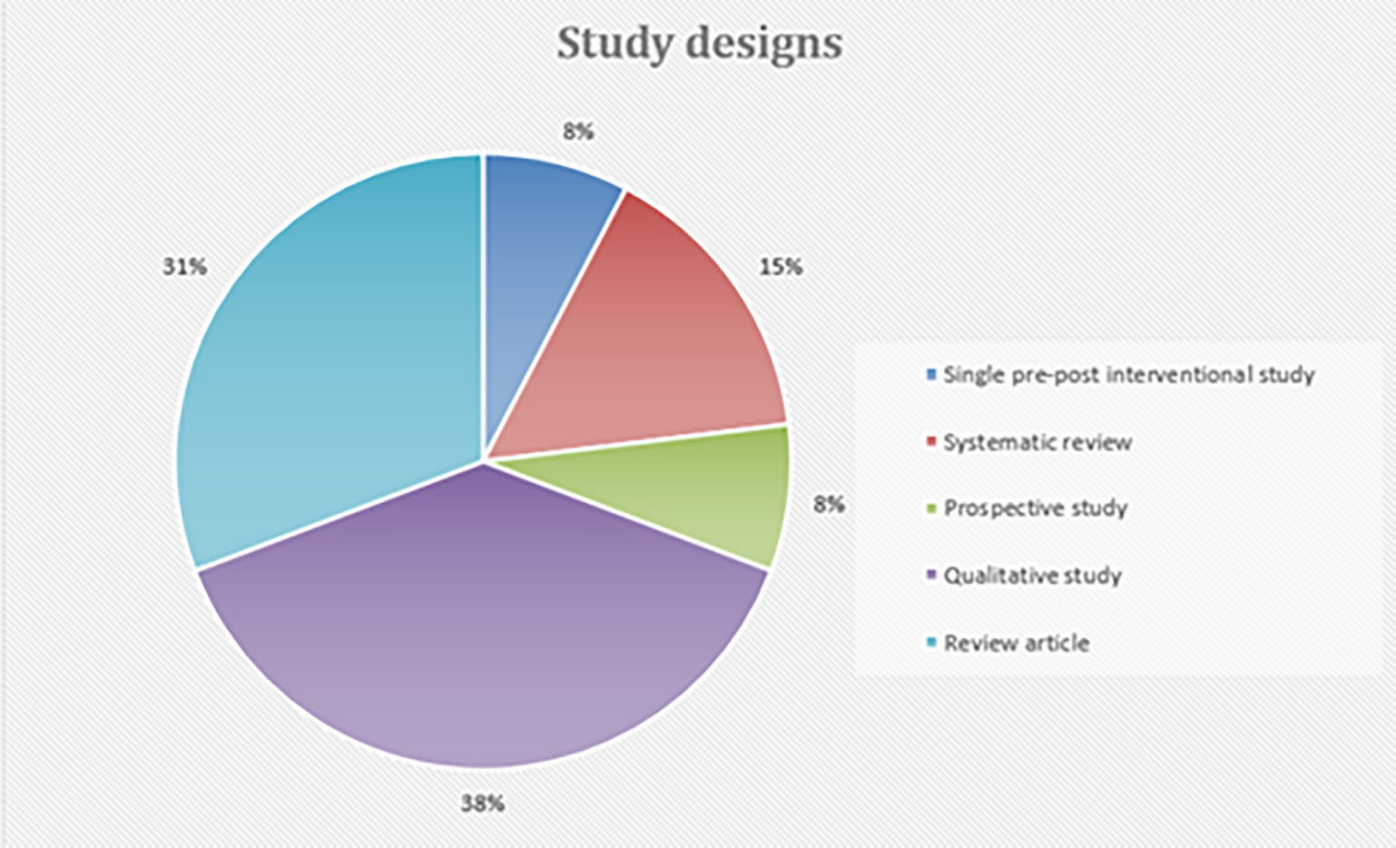
Demographic characteristics for study designs.

A total of 13 articles were included in the scoping review. ( Table 1– 3) There was 1 single group intervention pre-post pilot study design, ^28^ 2 systematic reviews, ^29^^,^^30^ 1 non-RCT (prospective study), ^31^ 5 qualitative observational studies, ^32^^–^^36^ 4 review articles. ^37^^–^^40^ ( Figure 2)

**Figure 3.**
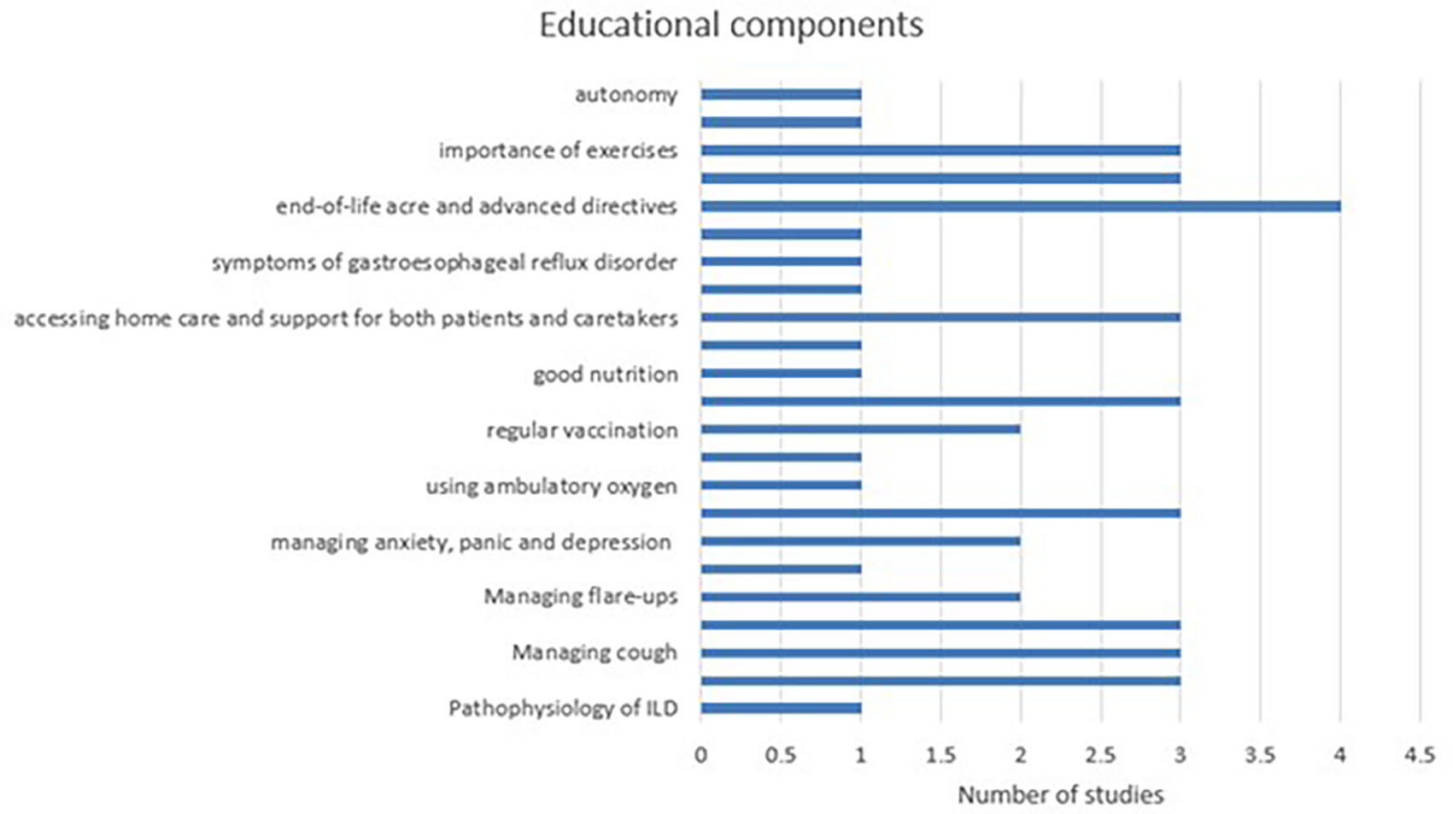
Components for patient education identified in literature for individuals with ILD.

All the studies described multiple PE topics. The most commonly discussed educational topics included: pathophysiology of ILD (1), ^35^ clinical tests (1), ^35^ managing breathlessness/dyspnoea (3), ^33^^,^^35^^,^^36^ managing cough (3), ^33^^,^^35^^,^^36^ managing fatigue (3), ^33^^,^^35^^,^^36^ managing flare-ups (2), ^33^^,^^36^ prognosis (1), ^35^ managing anxiety, panic and depression (2), ^33^^,^^36^ using supplemental oxygen (3), ^33^^,^^35^^,^^36^ using ambulatory oxygen (1), ^36^ using nocturnal oxygen (1), ^36^ regular vaccination (2), ^33^^,^^36^ managing medications and side-effects (3), ^33^^,^^35^^,^^36^ good nutrition (1), ^33^ managing co-existing medical condition (1), ^33^ accessing home care and support for both patients and caretakers (3), ^33^^,^^35^^,^^36^ smoking cessation (1), ^33^ symptoms of gastroesophageal reflux disorder, ^29^ adherence, ^29^ end-of-life acre and advanced directives (4), ^33^^,^^35^^,^^36^^,^^40^ valued education regarding pulmonary rehabilitation (3), ^32^^,^^33^^,^^34^ importance of exercises (3), ^32^^,^^33^^,^^34^ discussion over ILD specific topics (1), ^36^ autonomy (1). ^36^ ( Figure 3)

**Figure 4.**
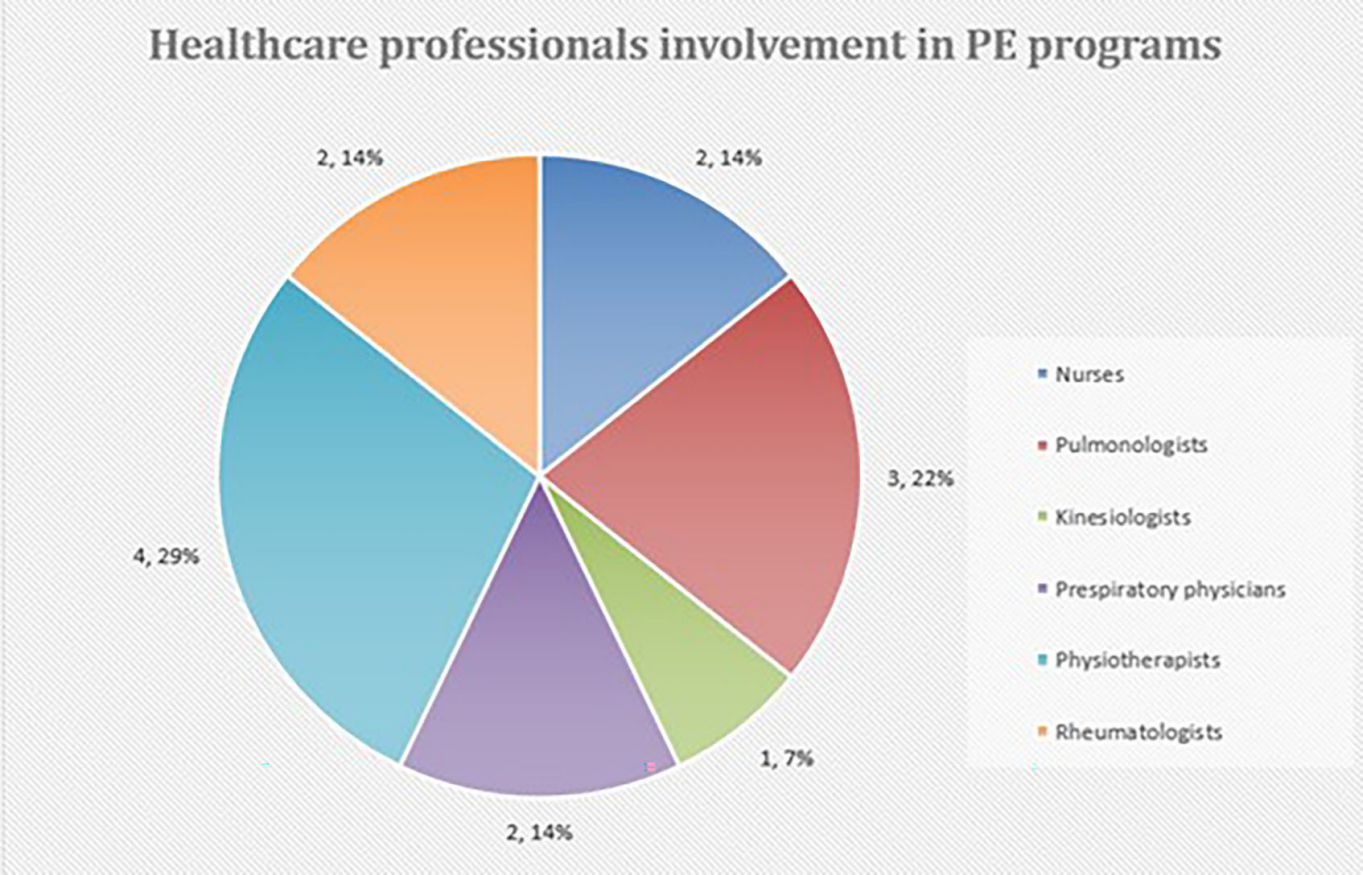
Involvement of healthcare professionals identified in PE programs for Individuals with ILD.

5 studies identified the involvement of health practitioners such as nurses (2) ^34^^,^^35^ pulmonologists (3), ^32^^,^^33^^,^^36^ kinesiologists (1), ^34^ respiratory physicians (2), ^32^^,^^36^ physiotherapists (4), ^29^^–^^32^ and rheumatologists (2), ^32^^,^^36^ in delivering PE to patients with ILD. A few studies (3) also mentioned the need for involvement of bystanders and caregivers in the PE sessions along with patients with ILD. ^28^^–^^30^ ( Figure 4)

*Process of delivery:*

PE was found to be implemented in a variety of settings including outpatient settings, outpatient supervised PR programs, inpatient PR programs, primary healthcare centers, and acute care units. ^29^^–^^31^ However, none of the studies mentioned use of PE as a part of the treatment program for ILD patients. ^29^^–^^31^

*Qualitative findings:*

The majority of the articles included in the scoping review were qualitative study designs. ^32^^–^^36^ These study designs aimed at highlighting the core topics and themes required for the development of educational booklets concerning the patient point of view. ^32^^,^^33^ These studies describe the interests of the patients, their understanding about the disease and brought to light their expectations from the physicians and healthcare professionals with respect to the disease by qualitatively analyzing their perspectives via focused groups and in-depth interviews. ^32^^–^^35^

Most of the qualitative studies were conducted either by a face-to-face communication, ^32^^–^^35^ via a telephonic interview, ^33^^,^^34^ website surveys, ^36^ questionnaire prescription ^34^ to design the components of PE in ILD for patient centred approach. Interviews were conducted with patients to collect sociodemographic information; this information included their management of ILD (e.g., drug therapy, specialist input, acute ILD exacerbations), satisfaction with the overall handling of their disease, medication profile, and travel costs. ^32^^–^^36^ Holland AE et al., in a qualitative review enlisting core topics essential for inclusion in the PE program for ILD patients stating the need for ILD specific PE packages for patients. ^33^

*Delivery technique:*

A few studies mentioned prescribing PE orally as a part of the rehabilitation or treatment program, although content was not disclosed. ^30^^,^^31^ A few mentioned the use of websites such as Pulmonary Fibrosis Foundation ( www.pulmonaryfibrosis.org), ( https://www.ofev.com/support/open-doors; https://www.esbriethcp.com/ipf-patient-assistance/patient-education-inspiration.html), web-based information booklet ^28^ for delivering PE among ILD patients and caregivers specifically. ^37^

*Effectiveness (Outcome measures):*

None of the articles used PE-specific outcome measures. But for their overall study objectives, they used different outcomes measures. ^28^^,^^29^^,^^30^^,^^34^

*Quality of life:*

Niemitz M et al., (2019) used self-reported scales to rate self-efficacy, scales for patients and caregivers to rate health-related quality of life (chILD-QOL), satisfaction with PE. ^28^ The majority of the studies used disease-specific, health-related quality of life tools as an outcome measure. Scales such as St. George Respiratory Questionnaire (SGRQ), ^29^^,^^30^ Chronic Respiratory Disease Questionnaire (CRQ), ^29^^,^^30^ Short Form- 36 (SF-36), ^29^^,^^30^ King’s brief Interstitial Lung Disease questionnaire (K-BILD), ^35^ University of California, Shortness of Breath Questionnaire (UCSD SOBQ), ^35^ International Physical Activity Questionnaire (IPAQ), ^30^ A Tool to Assess Quality of Life in Idiopathic Pulmonary Fibrosis (ATAQ IPF) scores, ^30^ Idiopathic Pulmonary Fibrosis (IPF) specific SGRQ scores. ^30^

*Physical status, dyspnoea, lung function:*

Authors have used indicators such as 6 MWD to determine ILD patients' physical status. ^30^ For dyspnea stages, Beck’s Depression Inventory (BDI), ^30^ and Modified Borg Scale (MBS) ^31^ were used. For the assessment of performance indicators, spirometric indicators like Forced Vital Capacity (FVC), Forced Expiratory Reserved Volume in 1 Second (FEV1) and diffusing capacity of lung of carbon monoxide (DLCO) were used. ^30^

In patients with ILD, PE is an integral component of care. Before beginning, a physiotherapist has an overall objective or goal for PE. The learning objectives for knowledge acquisition, solving problems, learning skills and behavioural change can be helpful. The method used could be visual and realistic, including demonstrations. ^29^^,^^30^ The physiotherapist would evaluate when and where to incorporate PE during rehabilitation. ^32^^,^^34^^–^^36^

## 4. Discussion

### About the disease

In line with the research questions laid down by us in our review, the first question focuses on the contents (topics) of PE provided for patients living with ILD. Patients require healthcare professionals to devote time with them after diagnosis, as highlighted by the studies included in this scoping review. ^29^^,^^30^^,^^40^ The patients with ILD should be given with a brief introduction regarding the disease and its course. They should be provided with an insight of what happens to the lungs because of the disease, various triggers, irritants and causes of ILD. They should be given a brief knowledge about the signs and symptoms, situations during exacerbations and how to manage them. ^29^^,^^30^^,^^32^^,^^40^

### Management

A multidisciplinary approach towards treating ILD patients is of utmost importance concerning managing the disease course. This involves the participation of the healthcare team, the patient themselves, and their caregivers. ^28^^,^^29^^,^^36^^,^^39^ PE must include awareness regarding the medications prescribed their effects on the body systems. Reactive oxygen species are responsible for tissue damage in ILDs, despite the fact that oxygen deficiency also occurs in these circumstances. Recognizing the significance of reactive oxygen species offers novel opportunities for therapeutic intervention.

Reference 41 Supplemental oxygen is one such component the patient should be made aware about. Since patients frequently receive oxygen therapy at home, the findings in this review recommend that patients should obtain PE regarding oxygen dosage and concentration during exacerbations and before engaging in strenuous activities. A large subgroup of people with ILD are normoxic at rest but rapidly desaturate on exertion. This can limit exercise capacity and worsen dyspnoea. When mobilizing or doing other tasks, using ambulatory or short-burst oxygen may help enhance exercise capacity and relieve dyspnoea. ^43^ Including a discussion of these concepts within the PE approaches both via face-to-face discussions by the pulmonologist, rheumatologist, immunologist, physiotherapist, and the staff nurses and reiterated in a written form using booklets or flyers is essential. Oxidative stress, which results from exposure to oxidizing agents including cigarette smoke, air pollution, and infections, has been linked to a variety of lung conditions. Therefore, the onset or advancement of these diseases has been linked to dietary components and nutrients that may have protective effects against oxidative processes and inflammatory reactions.

A dietician should be involved for discussion regarding the various areas the patients can reach out to and maintain and help establish an individualized diet plan for better living.

Exercise intolerance is an essential feature of ILD associated with poor quality of life and closely linked to mortality. Given the well-documented short-term effects of PR on lung function, quality of life, and improvements in activities of daily living, PE resources must emphasize the necessity of assessment and the advantages of exercise throughout the disease's course. Dysnoea and fatigue are key debilitating factors experienced by patients with ILD. The physiotherapist should spend time with the patients in educating them about the evaluation of the factors causing dyspnoea and fatigue and the non-pharmacological solutions to those problems which eventually affect the ADL’s and functional abilities of ILD patients. ^32^^,^^34^^,^^35^^,^^36^ In the studies included in this review, there must be a possible overlap between PR providers, which is likely to lack specificity.

There is lack of clarity regarding the contents of PE delivered to the patients with ILD by the physiotherapists. Individual perception of the patients, such as dyspnea, depression, and health-related quality of life (HRQoL), is more strongly interrelated. Consequently, the management of dyspnea and depressive symptoms could be an essential tool for optimizing the HRQoL of patients with ILD. Self-care is one such component that needs to be addressed to overcome exacerbations. Patients need to be briefed about the various techniques that can be used to self-manage their symptoms when in times of need. ^11^ There is no clear perspective in the current literature regarding the management of mental health among ILD patients. There is a lack of evidence and clarity regarding who provided the PE to the ILD patients and the duration spent doing the same in literature. Also, the effect of PE post-intervention on follow-up has not been addressed in any of the available literature. Outcome assessment tools need to be established for PE in ILD to know the knowledge of PE retained by the patients and its effects on their quality of life during follow-up.

Process of delivery:

In line with the research questions laid down by this review, the second question focuses on processes or methods used when providing PE to patients living with ILD. According to the content gathered from literature, various modalities of PE delivery to ILD patients can be used. There is lack of clarity regarding the process of PE delivery among ILD patients.

As of other chronic respiratory diseases, PE in ILD can be delivered verbally by the multidisciplinary healthcare team during their follow-up visits. ^30^^,^^31^^,^^44^^,^^45^ PE delivery through cutting-edge technology such as telerehabilitation allows the entire multidisciplinary team to collaborate and discuss the patient's whereabouts and caregiver involvement. ^46^

In contrast to other chronic respiratory disorders, PE can be delivered in ILD using a web-based platform that employs an innovative and digital design. ^28^^,^^37^ To communicate the contents of PE, healthcare practitioners can use instructional manuals, brochures, handouts, and other materials. ^29^^,^^30^ This can make the process easier as it becomes convenient for the patients and refer to the contents whenever possible. Educational booklets and handouts can also be a fantastic resource for disseminating PE knowledge in a diverse country like India, where there is a shortage of social-based platforms and smartphone use among individuals living in rural and remote sections of the country where internet penetration is very low. Educational booklets can be made exciting and understandable to the local population with the optimal use of illustrations, making it self-explanatory. By include progress/log charts in the booklet, we can keep track of adherence. Booklets can be a source to track, evaluate, and grade the performance for further progression.

### Limitations

During this scoping review, we identified significant diversity in the themes. The systematic reviews were unable to address the complexities of PE, limiting their capacity to give meaningful, evidence-based recommendations for PE. Also, a systematic review on educational programs in ILD management did not address PE topics with ILD patients. ^30^ While existing recommendations recognize crucial educational topics such as chronic pulmonary disease, dyspnea treatment, fatigue, oxygen therapy, and smoking cessation, none of these themes have been investigated in PE intervention clinical trials. ^29^^,^^30^^,^^40^ The study's limitations also included specific PE topics that were often not recognized clearly by a physiotherapist. According to this scoping review, the healthcare team, including pulmonologists, physiotherapists, psychologists, nurses, rheumatologists, and nutritionists, was all involved in delivering PE to ILD patients. ^30^^,^^32^^–^^36^ None of the studies gave details about the type of training obtained by physiotherapists and other healthcare professionals to provide effective PE. Whether health practitioners had been trained in PE and the study authors chose not to provide this knowledge in the paper or whether the clinicians had been trained in PE is unknown. Holland AE has enlisted the physician-centered perspective regarding core topics for PE prescription in her review. ^32^ The review discusses in detail the opinions of the patients and physicians regarding PE among ILD patients. She concluded that ILD patients appreciate the ability to undergo PR training, but they identify specific topics that do not currently cover their needs. Patients and clinicians did not agree upon the most important topics for inclusion. The construction of a comprehensive ILD PR curriculum should consider utilizing patients' knowledge and clinician approaches. ^32^

A couple of studies involved family members and caregivers with the patients themselves during the PE sessions. ^28^ Caregivers must be included in PE sessions to better grasp the disease process and help their loved ones emotionally. ^28^^,^^29^^,^^36^^,^^40^

The scoping review revealed that none of the studies addressed the location of PE delivery and follow-up periods for the educational sessions. The lack of recommendations on where and when to deliver PE for patients with ILD leads to this disparity. This is another significant gap in the literature.

The goals and priorities defined in the scoping review included PE but was not the primary intervention in the included studies. ^30^ The important findings were the cost-effectiveness of using healthcare facilities, exercise tolerance, quality of life, ^29^^,^^30^ and coping mechanisms. ^33^ The majority of research have classified PE as a self-management program, ^30^ however because the authors have not defined PE, explaining it as a self-management program is regrettably unclear. Self-management can imply different things to different people, and it's rarely defined in the literature. Some consider it synonymous with PE; others see PE as part of self-management.

The authors used a variety of PE delivery techniques and procedures, which can be divided into two categories: verbal and written approaches. ^30^^,^^31^ The authors have addressed education topic with ILD patients, who are by far the most common recipients of PE. ^30^ None of the studies mentioned the PE delivery mechanism or methodology. Healthcare practitioners, especially physiotherapists, employ various treatment strategies in PE, including the method of teaching that is undecisive. ^32^^,^^33^^,^^34^ Depending on the goals of PE, additional verbal methods can be further suited to adopt a team - based approach or a counselling approach. ^47^

Another common form of PE was giving patients booklets, ^31^ which were typically given in addition to discussion. However, they did not provide specific information on the contents of the booklet. This is also an efficacious method of PE delivery but must be easily understood irrespective of the level of literacy of the patient. ^48^^–^^55^ Few studies addressed the use for patients and caregivers of ILD-specific PE based on the web. ^28^ In future research, the emphasis should be on developing a patient disclosure guideline by ensuring that the print content is designed in plain language and simple design, ensuring consistency in PE.

The studies mainly used measures such as health-related quality of life questionnaires ^28^^–^^30^ and physiological performance measures as likely markers of PE efficacy. None of the studies have used outcomes measures explicitly to determine the effectiveness of PE. None concentrated on the influence of the PE alone. In addition, there are a limited range of valid PE instruments for patients with chronic diseases, while other methods are valid, reproducible, and sensitive to determine health status, quality of life ^28^^–^^30^ and physiological function and are well-established. One study discussed using self-reported scales to assess self-efficacy, quality of life and patient satisfaction for children with ILD. ^28^ Due to the heavy dependency on verbal contact in health care meetings, clear verbal communication, especially in PE, is essential for all health professionals. A 'Teach Back' informal approach for assessing the efficacy of PE ensures patients understand the directions. Future research could concentrate on those relevant methods that a health provider and a patient both settled on, for assessment of the efficacy of PE intervention with a PE specific goal, for example, information development, acquisition of skills, or behavioural change. ^41^^,^^42^

None of the studies have recorded disease and mortality, time for the first admission, number of hospital admissions and readmissions, reason for admission and overall hospital stays of days, appointments with the general practitioner and doctor visits, and prescription dispensations.

## 5. Conclusion

The aim of this scoping review was to examine and summarize the most recent information about the content, methods, and overall efficacy of patient education (PE) given to individuals with ILD. Our review revealed that providing PE as part of ILD care requires physiotherapists alongside other medical professionals. However, there is insufficient details about the precise material and procedures used by these professionals. Precise explanations of the educational subjects addressed, the environment, goals, and techniques for PE delivery for individuals with ILD are lacking in the literature. The absence of consistent, evidence-based recommendations on educational materials specifically designed for patients with ILD may be the cause of this ambiguity, given the variability of the studies that have been observed.

Also, the duration of PE provided or outcome measures to determine the PE's effectiveness is not specified. Further studies are needed to identify best practices for both the content and the processes of PE interventions and thereby optimize the quality of life of ILD patients.

## 6. Relevance to clinical practice

This scoping study will enable researchers to develop a patient-centered education manual/website that may be an instructional guide for delivering PE to ILD patients. Patients can get personalized help and better understand their disease, diagnosis, signs and symptoms, medications, coping methods, and pulmonary recovery (exercises and progression). The pulmonary rehabilitation education manual will act as a source of reinforcement and will assist in maintaining pulmonary rehabilitation compliance.

## Ethical approval

Ethical approval is not required for the study.

## Consent

Not applicable.

## Author contributions

K.V: Conceptualization and design of the study, acquisition of data, analysis and interpretation of data; R.A: Drafting the article, design of the study, acquisition of data, analysis and interpretation of data; G.A.M, A.K.M, M.K.S, A. B, T. B, V. A and S.G: Revising the manuscript critically for important intellectual content and provided final approval of the version to be submitted.

## Data Availability

No data are associated with this article. The author(s) own the figures attached below accompanying this manuscript. The author(s) declare that all the figures are their original work and not been adopted/reused from any other article. **Harvard Dataverse: Figure for** < Figure 1 Consort flow diagram>, https://doi.org/10.7910/DVN/MSNWBL. ^56^ **Harvard Dataverse: Figure for <** Figure 2 Demographic characteristics for study designs>, https://doi.org/10.7910/DVN/AW9AGO. ^57^ **Harvard Dataverse: Figure for <** Figure 3 Components for patient education identified in literature for individuals with ILD>, https://doi.org/10.7910/DVN/SGHSH5. ^58^ **Harvard Dataverse: Figure for <** Figure 4 Involvement of healthcare professionals identified in PE programs for Individuals with ILD>, https://doi.org/10.7910/DVN/TOJYWC. ^59^ Data are available under the terms of the Creative Commons Zero “No rights reserved” data waiver (CC0 1.0 Public domain dedication). 10/DVN/TOJYWC. **Harvard Dataverse: Checklist for <** Reporting Guidelines: Preferred Reporting Items for Systematic reviews and Meta-Analyses extension for Scoping Reviews (PRISMA-ScR) Checklist>, https://doi.org/10.7910/DVN/RXDIMN
