# Supplementary material for: Patient education for individuals with Interstitial Lung Disease: A scoping review
Source: F1000Res. 2024 Apr 26;13:405. [Version 1] doi: 10.12688/f1000research.147340.1 (PMC11184280; doi:10.12688/f1000research.147340.1)
Supplement: Peer review history

# Reviewer report: Reviewer response for version 1

**DOI:** 10.5256/f1000research.161527.r274667

**Published:** 2024-06-17

**Authors:** Sanjay T Parmar

**Affiliations:** 1 SDM College of Physiotherapy, Dharwad, India

1. The justification for performing a scoping review to map the existing knowledge gaps in PE for patients with ILD is explained in the introduction. You could explain on why previous studies on PE for ILD patients is insufficient or inadequate, emphasizing the necessity for additional study.
2. The introduction discusses the lack of studies on the content, delivery, and efficacy of PE for ILD patients.
  To highlight the need of filling this knowledge gap, you could briefly discuss how it might affect clinical practice or patient outcomes.
3. There is discussion of a transparent method for selecting and screening articles, which includes independent evaluation by multiple reviewers.
  You could discuss any steps performed, such inter-rater reliability tests or calibration exercises, to guarantee consistency and reliability amongst reviewers.
4. The study emphasizes critical elements such medication management, oxygen therapy, nutrition, and exercise and acknowledges the significance of a multidisciplinary approach in the management of ILD.
  You may be more specific about how each member of the healthcare team contributes to patient education and care coordination.
5. Some terms or concepts mentioned, such as "reactive oxygen species" and  "telerehabilitation" may need further explanation.
6. The conclusions and implications of the review should include combining the major themes that were covered throughout, such as the importance of outcome measures, the lack of standardized approaches to PE delivery.

Are the rationale for, and objectives of, the Systematic Review clearly stated?

Yes

Is the statistical analysis and its interpretation appropriate?

Yes

If this is a Living Systematic Review, is the ‘living’ method appropriate and is the search schedule clearly defined and justified? (‘Living Systematic Review’ or a variation of this term should be included in the title.)

Yes

Are sufficient details of the methods and analysis provided to allow replication by others?

Yes

Are the conclusions drawn adequately supported by the results presented in the review?

Yes

Reviewer Expertise:

NA

I confirm that I have read this submission and believe that I have an appropriate level of expertise to confirm that it is of an acceptable scientific standard.

# Reviewer report: Reviewer response for version 1

**DOI:** 10.5256/f1000research.161527.r274658

**Published:** 2024-05-21

**Authors:** Saima Zaki

**Affiliations:** 1 Centre for Physiotherapy and Rehabilitation Sciences, Jamia Millia Islamia, New Delhi, India

Title

 In your eligibility criteria, you have mentioned that Patient Education (PE) considered during Pulmonary Rehabilitation (PR) is being taken into account. Therefore, to accurately reflect the focus of your study, the title should explicitly indicate that it pertains to Patient Education during Pulmonary Rehabilitation. The current title, "Patient education for individuals with Interstitial Lung Disease: A scoping review," can be misleading as it suggests that PE was delivered during various interventions, not specifically PR.

 Suggested Title Revision:

 "Patient Education during Pulmonary Rehabilitation for Individuals with Interstitial Lung Disease: A Scoping Review"

 Abstract

 Comment 1: In the abstract, the authors define Interstitial Lung Disease (ILD) as "a severe and rapidly progressing disease with a high fatality rate." While this definition captures the severity of some forms of ILD, it is important to note that ILD encompasses a diverse group of lung disorders, each with varying degrees of severity and rates of progression.

 I recommend refining the definition to better reflect this diversity. A more accurate definition would acknowledge that while some types of ILD, such as Idiopathic Pulmonary Fibrosis (IPF), are indeed rapidly progressing and have a high fatality rate, other forms may progress more slowly and have different prognoses.

 Suggested revision:

 "Interstitial Lung Disease (ILD) refers to a group of lung disorders characterized by inflammation and scarring of the lung tissue, leading to impaired gas exchange. The progression and severity of ILD vary widely depending on the specific type, with some forms, such as Idiopathic Pulmonary Fibrosis (IPF), being rapidly progressive and associated with a high fatality rate, while others may progress more slowly."

 Comment 2: In method, it is suggested that to mention name of searched databases.

 Comment 3: In the second paragraph of the introduction, the transition to Pulmonary Rehabilitation (PR) is abrupt and disrupts the flow of the narrative.

 Comment 4:  In the last three paragraphs of the introduction, there are several significant statements made without any references to support them. Providing references for these statements is crucial to establish credibility and allow readers to verify the information. Here are the specific areas where references are needed:

 Pulmonary Rehabilitation (PR) and Chronic Obstructive Pulmonary Disease (COPD):

 "Since PR was developed and designated for patients with chronic obstructive pulmonary disease (COPD), which has a disease mechanism and symptoms that are fundamentally different from ILD."

 Reference needed to support the historical context and the distinction between COPD and ILD.

 PE Issues Defined for COPD Patients:

 "PE issues defined for patients with COPD, such as the physiological basis for exercise limitation, symptoms, treatment, disease severity, and outcomes, may not be relevant or significant for patients with other pulmonary disorders, such as ILD."

 References needed to support the differences in educational needs and issues between COPD and ILD patients.

 Content and Mechanisms of PE Provision:

 "Although healthcare practitioners routinely deliver PE verbally to patients with ILD, little is known about the actual content and mechanisms of this provision, as well as its overall efficacy."

 References needed to support the claim about the lack of knowledge regarding the content and mechanisms of PE provision for ILD patients.

 Need for Mapping Available Research:

 "There is a need to map the available research because there is a lack of evidence about the administration and content of PE delivered in ILD patients."

 References needed to justify the need for the scoping review based on the current gaps in the literature.

 To enhance the credibility and robustness of your manuscript, I strongly recommend including appropriate references to support these statements. This will not only strengthen your argument but also provide readers with sources for further reading.

 Results

 Comment 5: There appears to be a major discrepancy in your study's reporting. In the PRISMA flowchart, it is stated that 12,642 articles were included in the scoping review. However, in the results section, it is mentioned that a total of 13 articles were included in the review. This inconsistency needs to be addressed and clarified to maintain the credibility and accuracy of your study.

 Comment 6: There is a significant and unacceptable discrepancy between the narrative description of the study selection process and the PRISMA flowchart in your manuscript. In the narrative, it is mentioned that 355 articles were selected for full-text review. However, the PRISMA flowchart states that 12,642 articles were included in the scoping review. This is a major inconsistency that undermines the credibility and integrity of your scientific writing.

 Such a blunder in reporting the methodology is not acceptable in scientific literature and must be corrected immediately. Accurate and consistent reporting of the study selection process is crucial for the transparency and reproducibility of your research.

 Discussion

 Comment 7: The discussion section lacks a brief summary of the main findings of your review in the first paragraph. This summary is crucial as it provides readers with an immediate understanding of the key outcomes of your study. Furthermore, there is no mention of Patient Education (PE) during Pulmonary Rehabilitation (PR) throughout the discussion, which is a significant oversight given the focus of your review.

 In the management section, the text begins appropriately with a discussion on the multidisciplinary approach but abruptly shifts to discussing reactive oxygen species without proper context or references. This sudden transition disrupts the flow and coherence of the section. Additionally, the statements about reactive oxygen species are not supported by any references, which is a significant omission in a scientific manuscript.

Are the rationale for, and objectives of, the Systematic Review clearly stated?

No

Is the statistical analysis and its interpretation appropriate?

Partly

If this is a Living Systematic Review, is the ‘living’ method appropriate and is the search schedule clearly defined and justified? (‘Living Systematic Review’ or a variation of this term should be included in the title.)

No

Are sufficient details of the methods and analysis provided to allow replication by others?

No

Are the conclusions drawn adequately supported by the results presented in the review?

Partly

Reviewer Expertise:

Interstitial Lung Disease, Pulmonary Rehabilitation, Cardiac Autonomic Modulation, Cardiorespiratory fitness and Training, Metabolic Disorders, Lifestyle Disorders, Sports Rehabilitation, Clinical Research, Patient Education and Counseling, Quality of Life

I confirm that I have read this submission and believe that I have an appropriate level of expertise to confirm that it is of an acceptable scientific standard, however I have significant reservations, as outlined above.

# Reviewer report: Reviewer response for version 1

**DOI:** 10.5256/f1000research.161527.r274664

**Published:** 2024-05-10

**Authors:** Jaya Shanker Tedla

**Affiliations:** 1 Department of Medical Rehabilitation Sciences, College of Applied Medical Sciences, King Khalid University, Abha, Aseer Province, Saudi Arabia

1. The introduction explains the justification for performing a scoping review to map the existing knowledge gaps in PE for patients with ILD. You could explain why previous studies on PE for ILD patients are insufficient or inadequate, emphasizing the necessity for additional studies.

 2. The methodology includes a thorough explanation of the search strategy, including terms utilized and the search procedure. You could discuss any steps to reduce bias in the search strategy, including consulting with a librarian or examining gray literature sources.

 3. The methodology could explain how the data extraction forms categories were identified and whether they evolved due to initial analysis or pilot testing.

 4. You could have sections like "Contents of Patient Education," Management Strategies, Process of Delivery, Limitations, etc. In the discussion.

 5. One of the main limitations is the lack of recorded data on hospital admissions, disease and mortality, etc. However, future research should address the consequences of this paucity of data on the interpretation of PE effectiveness, as well as the

 the necessity for more extensive outcome assessment.

Are the rationale for, and objectives of, the Systematic Review clearly stated?

Yes

Is the statistical analysis and its interpretation appropriate?

Not applicable

If this is a Living Systematic Review, is the ‘living’ method appropriate and is the search schedule clearly defined and justified? (‘Living Systematic Review’ or a variation of this term should be included in the title.)

Not applicable

Are sufficient details of the methods and analysis provided to allow replication by others?

Yes

Are the conclusions drawn adequately supported by the results presented in the review?

Yes

Reviewer Expertise:

Rehabilitation, Physical Therapy, Physiotherapy, Patient education, and Student teaching.

I confirm that I have read this submission and believe that I have an appropriate level of expertise to confirm that it is of an acceptable scientific standard, however I have significant reservations, as outlined above.
